# Intracellular coexpression of CXC- and CC– chemokine receptors and their ligands in human melanoma cell lines and dynamic variations after xenotransplantation

**DOI:** 10.1186/1471-2407-14-118

**Published:** 2014-02-22

**Authors:** Sandra Pinto, Alicia Martínez-Romero, José-Enrique O’Connor, Rosario Gil-Benso, Teresa San-Miguel, Liria Terrádez, Carlos Monteagudo, Robert C Callaghan

**Affiliations:** 1 Cytomics Laboratory, Mixed Unit CIPF-UVEG, Príncipe Felipe Research Centre, Valencia, Avda Autopista del Saler, 16, 46012 Valencia, Spain; 2 Current address: CNC-Center for Neurosciences and Cell Biology, University of Coimbra, 3004-517 Coimbra Portugal; 3 Department of Pathology, University of Valencia, Avda. Blasco Ibañez 15, 46010 Valencia Spain; 4 Department of Pathology, University Clinic Hospital INCLIVA, Avda. Blasco Ibañez 17, 46010 Valencia, Spain

**Keywords:** Melanoma, Cell line, Chemokine receptor, Chemokine, Xenotransplantation

## Abstract

**Background:**

Chemokines have been implicated in tumor progression and metastasis. In melanoma, chemokine receptors have been implicated in organ selective metastasis by regulating processes such as chemoattraction, adhesion and survival.

**Methods:**

In this study we have analyzed, using flow cytometry, the systems formed by the chemokine receptors CXCR3, CXCR4, CXCR7, CCR7 and CCR10 and their ligands in thirteen human melanoma cell lines (five established from primary tumors and eight established from metastasis from different tissues). WM-115 and WM-266.4 melanoma cell lines (obtained from a primary and a metastatic melanoma respectively) were xenografted in nude mice and the tumors and cell lines derived from them were also analyzed.

**Results:**

Our results show that the melanoma cell lines do not express or express in a low degree the chemokine receptors on their cell surface. However, melanoma cell lines show intracellular expression of all the aforementioned receptors and most of their respective ligands. When analyzing the xenografts and the cell lines obtained from them we found variations in the intracellular expression of chemokines and chemokine receptors that differed between the primary and metastatic cell lines. However, as well as in the original cell lines, minute or no expression of the chemokine receptors was observed at the cell surface.

**Conclusions:**

Coexpression of chemokine receptors and their ligands was found in human melanoma cell lines. However, this expression is intracellular and receptors are not found at the cell membrane nor chemokines are secreted to the cell medium. The levels of expressed chemokine receptors and their ligands show dynamic variations after xenotransplantation that differ depending on the origin of the cell line (from primary tumor or from metastasis).

## Background

Chemokines are chemoattractant cytokines which were initially characterized by their important role in leukocyte recruitment during inflammatory and immune responses [1]. They act through the interaction with a subfamily of G protein-coupled receptors [2] and the extent of the cellular response triggered by chemokines depends on the amount of receptor expressed at the plasma membrane, which is a consequence of the balance between endocytic and recycling pathways [3]. Several studies have shown that chemokines and their receptors are involved in different steps of tumorigenesis, including angiogenesis, tumor growth, invasion and metastasis of many human cancers [4,5].

Chemokines and their receptors are of great importance in the melanoma tumor progression [6]. The expression of CXCR4 by melanoma cells in primary lesions is significantly associated with the presence of ulceration, increased tumor thickness and higher mortality rate [7]. The expression of CXCR3 has been observed in patients with primary invasive cutaneous melanomas and there is a significant association of CXCR3-positive tumor cell immunostaining with tumor thickness >1 mm [8].

Cancer metastasis is the complex process by which primary tumors spread to a distant location and is the main cause of death for most cancer patients [9]. Chemokines and their receptors regulate organ selectivity in metastasis. They are expressed at specific organs and act on tumor cells, which express the correspondent receptors, inducing their directed migration. Chemokines also improve tumor cell proliferation, survival and adhesion to microvessel walls, helping the process of extravasation into the target tissue [10,11]. Indeed, it has been shown that mouse melanoma B16F10 cells constitutively express CXCR3, and its ligands CXCL9/Mig, CXCL10/IP-10, and CXCL11/I-TAC induce cellular responses in vitro, such as actin polymerization, migration, invasion, and cell survival [12]. Moreover, the expression of several chemokine receptors has been associated with a greater risk of developing regional and distant metastases [7,8] as lymph node metastasis (CCR7 [13]; CCR10, [14]; CXCR3 and CXCR4; [15]), pulmonary metastasis (CXCR4) or skin metastasis (CCR10; [13]). The role in melanoma of the recently discovered CXCR7, which binds to CXCL11 and CXCL12 [16] is still not clear. However, it has been shown that CXCR7 is involved in tumor cell growth, survival, and metastasis in several tumor types [17].

The aim of this work was to characterize the secretion and the intracellular expression of the chemokines CXCL9, CXCL10, CXCL11, CXCL12, CCL19, CCL21, CCL27 and CCL28 and the surface and intracellular expression of their chemokine receptors CXCR3, CXCR4, CXCR7, CCR7 and CCR10 in human melanoma cell lines, and the effect of xenotransplantation on the chemokine/chemokine receptor expression. We have included cell lines of primary and metastatic origin, as differences in chemokine receptors expression have been reported in uveal melanoma cell lines depending on their origin [18].

## Methods

### Cell lines and cell culture conditions

The selected cell lines included cells of primary and metastatic origin. Thirteen human melanoma cell lines were used (Table 1), five established from primary tumors and eight established from metastases at different locations. Twelve of these cell lines are commercially available and one (Mel-RC08) was established and characterized in our laboratory [19]. The Hut-78 cell line was also included in this study as it was previously reported that it expresses some of the chemokine receptors studied [20]. All cell lines were grown in a humidified atmosphere with 5% CO_2_ using RPMI 1640 medium, supplemented with 10% fetal bovine serum inactivated by heat, 2 mM L-glutamine, 100 UI/mL penicillin and 100 μg/mL streptomycin sulfate (Gibco™, Invitrogen, Carlsbad, CA, USA).

**Table 1:** Cell lines

***Tumor***	***Cell line***	***Origin***	***Source***
Melanoma	IPC-298	Primary	DSMZ
	Mel-Juso	Primary	DSMZ
	Mel-HO	Primary	DSMZ
	IGR-39	Primary	DSMZ
	WM-115	Primary	ECACC
	A-375	Skin metastasis	ATCC
	MeWo	Lymph node metastasis	ATCC
	SK-Mel28	Skin metastasis	ATCC
	Malme-3 M	Lung metastasis	ATCC
	SK-Mel 2	Skin metastasis	ATCC
	WM-266.4	Skin metastasis^(1)^	ECACC
	IGR-37	Lymph node metastasis ^(2)^	DSMZ
	Mel-RC08	Brain metastasis	DPUV
Human T-cell Lymphoma	Hut-78	Primary	ECACC

List of tested cell lines, tumor type, origin (primary or metastasis) and source. DSMZ – German Resource Centre for Biological Material, ECACC – European Collection of Cell Cultures, ATCC - American Type Culture Collection, DPUV – Department of Pathology, University of Valencia, Valencia, Spain).

^(1)^Established from the same patient as cell line WM-115.

^(2)^Established from the same patient as cell line IGR-39.

### Human tumor xenografts and derived cell lines

The primary WM-115 and the metastatic WM-266.4 cell lines, established from the same patient, were inoculated into 4–6 weeks old BALB/c athymic nude mice (Charles River, Spain). Each mouse was inoculated with 200 μl containing 2×10^6^ cells, and due to different growth rates of tumors, the animals inoculated with WM-266.4 were sacrificed 35 days after inoculation and the ones inoculated with WM-115 were sacrificed 77 days after inoculation. All procedures were performed according to the institutional recommendations and guidelines for the good care of laboratory animals, and were approved by the Research Ethical Committee of the University Clinic Hospital-INCLIVA, Valencia.

Tumors were extracted and disaggregated using 0.02% collagenase type II (Sigma-Aldrich, St. Louis, MO, USA). Cells were resuspended in culture medium and part of them were immediately used in flow cytometry experiments (hereafter referred as xenografts WM-115-X and WM-266-X) and some cells were maintained in culture to establish new cell lines after xenotransplantation. Five cell lines were obtained from different xenografts of WM-115 and six cell lines were derived from distinct xenografts of WM-266.4. These modified cell lines were named WM-115-CX and WM-266-CX (CX stands for cultured xenografts).

### Immunocyochemistry

Cells were grown in Lab-Tek chamber slides (Miles Laboratories, Naperville, IL, USA). After washing with PBS, the cells were fixed with cold methanol-acetone for 5 min. Mouse monoclonal antibodies (mAb) against human CXCR3, CXCR4, CXCR7, CCR7, CXCL12, CCL19, CCL27 were from R&D Systems (Minneapolis, MN, USA) and mouse mAb against human CXCL10 was from BD Biosciences (Franklin Lakes, NJ, USA). Goat anti-human CCR10 polyclonal antibody (pAb) was from Abcam (Cambridge, MA, USA), goat anti-human CXCL9 and CCL21 pAbs were from R&D Systems and rabbit anti-human CXCL11 pAb was from Peprotech (Rocky Hill, NJ, USA). The cells reacted with each of these primary antibodies for 1 h at room temperature. The attached antibodies were visualized by the avidin-biotin-peroxidase procedure (Dako, Carpentaria, CA, USA).

### Flow cytometry

#### Antibodies and fluorochromes

All mAbs against chemokine receptors were conjugated to phycoerytrin (PE). mAbs against CXCR3, CXCR4, CCR7 and correspondent isotypic controls were purchased from BD Biosciences. mAbs against CXCR7 and CCR10 and correspondent isotypic controls were purchased from R&D Systems. All chemokine ligands (CXCL9, CXL10, CXCL11, CXCL12, CCL19, CCL21, CCL27 and CCL28) were detected using primary mouse-anti-human mAbs from R&D Systems with a secondary goat anti-mouse antibody labeled with FITC (R&D Systems). The DNA intercalating fluorochrome 7-Aminoactinomycin D (7-AAD, Sigma-Aldrich) was used for dead cells staining.

#### Cell surface expression of chemokine receptors

Cells at subconfluency (50–70%) were detached with 2 mM EDTA in PBS, washed and resuspended in ice-cold culture medium at 1×10^6^cells/ml (the suspension cell line Hut-78 was also resuspended at the same concentration). Subsequently, 100 μl of this cell suspension were incubated on ice for 30 min with the chemokine receptors mAbs and correspondent isotypic controls. After incubation, cells were washed with ice-cold PBS and resuspended in 500 μl of culture medium for flow cytometric analysis. To determine dead cells 3 μg/ml 7-ADD were added prior to cytometric analysis. The mean percentage (%) of cells which expressed chemokine receptors at the cell surface, as well as the mean fluorescence intensity (MFI) calculated as the ratio between the mean fluorescence of the positive population in the samples stained with the mAb anti-receptor and the correspondent isotypic control were determined.

#### Intracellular expression of chemokine receptors

Cells at subconfluency (50–70%) were detached with 2 mM EDTA in PBS, washed, fixed with 4% paraformaldehyde and permeabilized with 0.1% Triton/PBS adjusting cell suspension to 1×10^6^cells/ml. Then, 100 μl volumes of this cell suspension were incubated on ice for 30 min with the chemokine receptors mAb and correspondent isotypic controls. After incubation, cells were washed with ice-cold 1%BSA/PBS and resuspended in 500 μl of ice-cold 1%BSA/PBS for flow cytometric analysis. The mean percentage (%) of cells which expressed chemokines intracellularly, as well as the MFI calculated as the ratio between the mean fluorescence of samples stained with the mAb anti-receptor and the correspondent isotypic control were determined.

#### Intracellular chemokine expression

Cells at subconfluency (50–70%) were detached with 2 mM EDTA in PBS, washed, fixed with 4% paraformaldehyde and permeabilized with 0.1% Triton/PBS adjusting cell suspension to 1×10^6^cells/ml. Subsequently, 100 μl volumes of this cell suspension were incubated on ice for 30 min with the chemokine unconjugated mAbs. Afterwards, cells were washed twice with ice-cold 1%BSA/PBS and incubated on ice for 30 min with the secondary FITC-conjugated antibody. An aliquot of 100 μl of cell suspension incubated only with the secondary FITC-conjugated antibody was used as a control for all the chemokine ligands. Finally, cells were washed twice and resuspended in 500 μl of ice-cold 1%BSA/PBS for flow cytometric analysis. The mean percentage (%) of cells which expressed chemokine receptors intracellularly, as well as the MFI calculated as the ratio between the mean fluorescence of samples stained with the mAb anti-receptor and the correspondent isotypic control were determined.

The quantification of cell surface expression of chemokine receptors and the intracellular expression of chemokines and their receptors was always performed in the same day, 24 h after sub-culturing, using cells with identical culture conditions.

#### Cytometer settings

All the analyses were performed in a FC500 MCL flow cytometer (Beckman-Coulter, CA, USA) with an air-cooled argon ion laser (488 nm, 15 mW). This standard instrument is equipped with two light scatter detectors that measure the forward scatter (an estimation of cell size) and the side scatter (an estimation of intracellular complexity), and five photomultiplier tubes that detect the appropriately filtered light. FITC fluorescence was collected at 525 ± 20 nm, PE at 575 ± 20 nm and 7-AAD at 675 ± 20 nm. When determining dead cells (for quantification of the cell surface expression of receptors) all measurements were restricted to live cells by gating the cells that excluded 7-AAD. In all other cases, the population was selected based on forward and side scatter parameters.

### Chemokine secretion

The quantification of chemokine secretion levels was performed in cell culture medium collected 24 hours after sub-culturing the cells using the commercial multiplex kits *MILLIPLEX™ Multi-Analyte Profiling (MAP)* (Millipore, Billerica, MA, USA) according to manufactures indications. Furthermore, as a positive control the secretion of IL-8 and Gro were also quantified. Cells were grown in 10 ml of culture medium and after 24 hours of sub-culturing reached approximately 70% confluency. The processed samples were subsequently analyzed using Luminex 100™ System (Luminex Coorporation, Austin, TX, USA).

### Statistical analysis

All measurements in cell lines were made in triplicate. For flow cytometry experiments, the number of positive cells stained with the different antibodies was compared with the number of positive cells in the correspondent negative controls (isotype or secondary antibody) and the differences were analyzed using Student’s t-test and considered significant when p < 0.05. For chemokine secretion experiments, the concentration obtained in each sample was compared to the lowest standard concentration of the standard curve and the differences were analyzed using Student’s t-test, and considered significant when p < 0.05. The comparison between the expression of chemokines and their receptors between the original cell lines WM-115 and WM-266.4 and the tumors (WM-115-X, WM-266-X) and cell lines (WM-115-CX, WM-266-CX) obtained after xenotransplantation was analyzed using Student’s t-test and considered significant when p < 0.05.

## Results

### Surface expression of chemokine receptors CXCR3, CXCR4, CXCR7, CCR7 and CCR10

**Figure 1:**
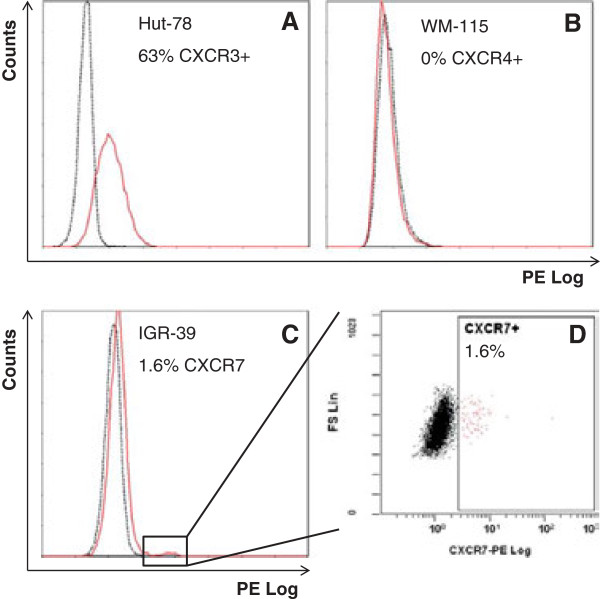
**Surface expression of chemokine receptors.** Representative examples for the quantification of chemokine receptors surface expression by flow cytometry are shown. Overlaid histograms of PE fluorescence of specific anti-receptor monoclonal antibody (continuous red line) and correspondent isotypic control (discontinuous black line) show the control Hut-78 cell line with a high percentage of CXCR3+ cells **(A)**, WM-115 with no expression of CXCR4 **(B)** and IGR-39 with a small subpopulation of CXCR7+ cells **(C)**, which is further illustrated in a biparametric dotplot of FS vs PE fluorescence **(D)**.

We found that melanoma cell lines did not express or express in a low degree (less than 2% of the population; Table 2) the chemokine receptors on their cell surface. The small positive subpopulations were mostly observed in lines obtained from primary tumors. Representative flow cytometry plots are shown in Figure 1.

**Table 2:** Surface expression of chemokine receptors

***Origin***	***Cell line***	***CXCR3***		***CXCR4***		***CXCR7***		***CCR7***		***CCR10***	
		***%***	***MFI***	***%***	***MFI***	***%***	***MFI***	***%***	***MFI***	***%***	***MFI***
P	IPC-298	0.16	3.10	ns	-	ns	-	ns	-	0.23	11.37
P	Mel-Juso	ns	-	ns	-	0.43	27.70	ns	-	0.43	12.74
P	Mel-HO	0.07	4.83	ns	-	0.36	4.91	ns	-	ns	-
P	IGR-39	0.83	8.31	ns	-	1.59	7.09	ns	-	1.79	4.05
P	WM-115	0.15	5.91	ns	-	ns	-	ns	-	0.11	5.09
M	A-375	ns	-	ns	-	ns	-	ns	-	ns	-
M	MeWo	ns	-	ns	-	ns	-	ns	-	ns	-
M	SK-Mel28	ns	-	ns	-	ns	-	ns	-	ns	-
M	Malme-3 M	ns	-	ns	-	ns	-	ns	-	ns	-
M	SK-Mel 2	0.44	3.16	ns	-	0.35	10.34	ns	-	0.35	3.84
M	WM-266-4	ns	-	ns	-	ns	-	ns	-	ns	-
M	IGR-37	0.13	3.47	ns	-	0.22	14.76	ns	-	ns	-
M	Mel-RC08	ns	-	ns	-	ns	-	ns	-	ns	-
P	Hut-78	63.07	3.37	5.90	6.91	ns	-	3.36	7.66	1.23	8.36

For each cell line (Origin; P: primary tumor, M: metastasis) mean percentage (%) and mean fluorescence index (MFI; calculated as the ratio between the mean fluorescence of the positive population in the samples stained with the mAb anti-receptor and their correspondent isotypic control) of cells which significantly expressed (p < 0.05) chemokine receptors at the cell surface are shown (ns: not significant expression).

### Intracellular expression of chemokine receptors CXCR3, CXCR4, CXCR7, CCR7 and CCR10 in human melanoma cell lines

**Figure 2:**
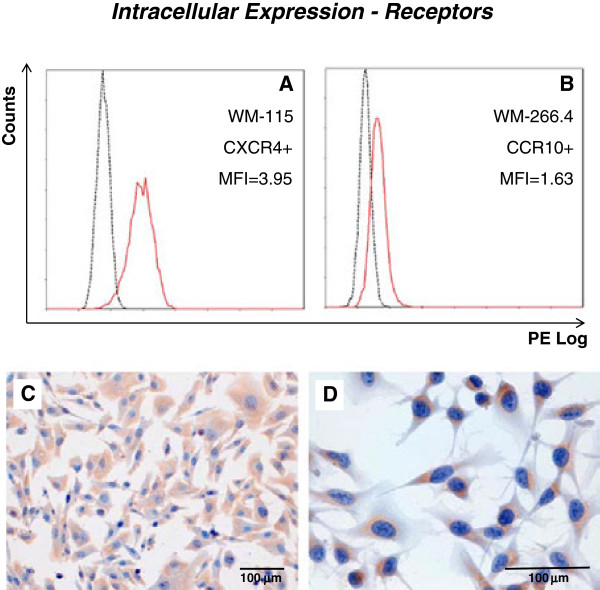
**Intracellular expression of chemokine receptors.** Representative examples for the quantification of intracellular chemokine receptor expression by both flow cytometry **(A, B)** and immunocytochemistry **(C, D)** are shown. Mean fluorescence indexes and overlaid histograms of PE fluorescence of specific anti-receptor monoclonal antibody (continuous red line) and correspondent isotypic control (discontinuous black line) are shown for CXCR4 in the WM-115 cell line **(A)** and for CCR10 in the WM-266.4 cell line **(B)**. Corresponding immunocytochemical staining of CXCR4 in WM-115 **(C)** and CCR10 in WM-266.4 **(D)**.

All cell lines significantly expressed all chemokine receptors intracellularly (Table 3). However, variability was found in the pattern of expression depending on the cell line and receptor studied. Representative histograms are shown in Figure 2A and B. Furthermore, the level of protein expression varied between receptors and cell lines. In general, CXCR4 seems to be the most expressed receptor, with higher MFI values, while CCR7 appears to be the receptor which is expressed at lowest levels, having lower MFI values (Table 3).

**Table 3:** Intracellular expression of chemokine receptors

***Origin***	***Cell line***	***CXCR3***		***CXCR4***		***CXCR7***		***CCR7***		***CCR10***	
		***%***	***MFI***	***%***	***MFI***	***%***	***MFI***	***%***	***MFI***	***%***	***MFI***
P	IPC-298	99.45	7.34	99.41	6.29	21.26	1.48	58.30	1.92	45.67	1.86
P	Mel-Juso	53.74	3.27	62.50	4.16	3.58	1.18	39.58	2.51	18.64	1.78
P	Mel-HO	96.71	5.28	98.99	7.82	53.07	2.21	82.49	3.13	56.36	2.26
P	IGR-39	95.73	3.73	95.76	4.04	81.82	2.59	87.87	2.43	37.51	1.70
P	WM-115	88.59	5.40	92.45	3.95	4.97	1.57	20.16	1.70	13.67	1.57
M	A-375	43.61	2.51	94.44	14.10	63.20	3.45	12.70	1.59	45.45	2.89
M	MeWo	25.02	2.21	80.60	5.98	80.65	6.59	2.58	1.32	61.27	3.53
M	SK-Mel28	56.56	2.43	93.22	8.63	73.86	5.02	1.20	1.20	65.81	2.59
M	Malme-3 M	64.99	2.07	92.84	2.66	97.78	5.66	64.86	1.99	71.88	2.37
M	SK-Mel 2	96.43	6.92	98.33	15.73	33.52	2.04	78.23	2.08	96.34	4.68
M	WM-266-4	74.08	2.22	96.26	3.39	21.53	1.68	40.16	1.69	24.00	1.63
M	IGR-37	97.88	4.25	97.85	4.53	43.84	1.89	67.83	2.03	56.29	1.89
M	Mel-RC08	45.79	2.69	95.02	7.33	72.05	3.99	9.14	1.61	60.81	2.84
P	Hut-78	82.05	2.93	95.95	4.62	19.75	1.95	13.53	1.68	88.40	4.08

For each cell line (Origin; P: primary tumor, M: metastasis) mean percentage (%) and mean fluorescence index (MFI; calculated as the ratio between the mean fluorescence of the samples stained with the mAb anti-receptor and their correspondent isotypic control) of cells which significantly expressed (p < 0.05) chemokine receptors intracellularly are shown (ns: not significant expression).

### Intracellular expression of chemokines CXCL9, CXCL10, CXCL11, CXCL12, CCL19, CCL21, CCL27 and CCL28 in human melanoma cell lines

**Figure 3:**
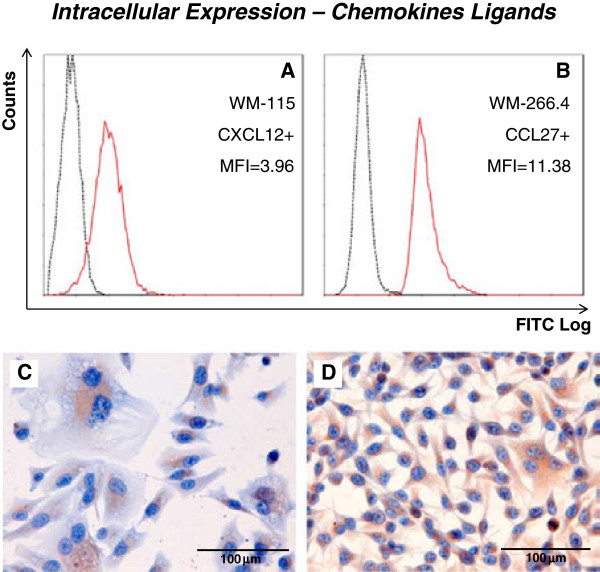
**Intracellular expression of chemokines.** Representative examples for the quantification of intracellular chemokine expression by both flow cytometry **(A, B)** and immunocytochemistry **(C, D)** are shown. Mean fluorescence indexes and overlaid histograms of PE fluorescence of specific anti-receptor monoclonal antibody (continuous red line) and correspondent isotypic control (discontinuous black line) are shown for CXCL12 in the WM-115 cell line **(A)** and for CCL27 in the WM-266.4 cell line **(B)**. Corresponding immunocytochemical staining of CXCL12 in WM-115 **(C)** and CCL27 in WM-266.4 **(D)**.

Most chemokines were expressed intracellularly in all melanoma cell lines (CXCL9, CXCL11, CXCL12, CCL19, CCL21 and CCL27). The chemokines exhibiting lower levels of intracellular expression were CXCL10 and CCL28 (Table 4). Representative histograms are shown in Figure 3A and B. The control Hut-78 cell line only expressed CXCL9, CCL19 and CCL27 significantly, and at lower levels than the melanoma cell lines.

**Table 4:** Intracellular expression of chemokine ligands

***Origin***	***Cell line***	***CXCL9***		***CXCL10***		***CXCL11***		***CXCL12***		***CCL19***		***CCL21***		***CCL27***		***CCL28***	
		***%***	***MFI***	***%***	***MFI***	***%***	***MFI***	***%***	***MFI***	***%***	***MFI***	***%***	***MFI***	***%***	***MFI***	***%***	***MFI***
P	IPC-298	95.39	3.69	0.93	1.11	7.00	1.36	97.93	5.34	99.02	16.86	53.98	1.94	98.71	7.39	ns	ns
P	Mel-Juso	93.22	10.88	ns	ns	12.90	2.10	89.57	5.80	99.04	70.62	6.12	1.60	98.75	17.36	ns	ns
P	Mel-HO	96.59	8.39	7.32	1.32	65.87	5.83	97.86	8.12	98.62	60.07	82.62	3.72	98.65	23.48	ns	ns
P	IGR-39	14.05	1.52	ns	ns	10.49	1.37	95.86	3.89	98.76	16.71	23.50	1.54	98.58	5.21	ns	ns
P	WM-115	28.31	2.06	0.46	1.12	2.66	1.24	81.77	3.96	98.79	20.00	15.55	1.65	94.06	4.89	ns	ns
M	A-375	84.56	6.18	0.42	1.07	88.71	7.46	10.89	1.67	92.34	71.31	32.60	3.42	89.92	25.21	1.14	1.29
M	MeWo	45.53	5.28	1.46	1.44	46.10	8.72	24.41	2.15	50.06	34.71	23.62	3.96	37.85	10.86	1.74	1.86
M	SK-Mel28	67.63	3.06	2.01	1.18	82.59	5.72	71.82	3.09	86.62	44.41	28.42	1.96	86.34	14.37	ns	ns
M	Malme-3 M	50.32	3.45	ns	ns	71.62	3.45	77.48	3.37	98.02	34.57	5.45	1.70	97.90	11.06	ns	ns
M	SK-Mel 2	81.72	3.18	ns	ns	94.63	5.53	95.94	8.46	98.34	36.70	22.45	1.86	97.47	13.02	ns	ns
M	WM-266-4	71.84	4.57	15.19	1.74	46.26	3.24	98.24	9.56	98.58	30.17	60.21	3.71	98.50	11.38	1.14	1.25
M	IGR-37	87.92	5.06	ns	ns	36.32	1.88	92.76	3.78	98.83	47.66	38.71	2.44	98.96	21.35	ns	ns
M	Mel-RC08	49.96	3.29	ns	ns	80.84	6.87	16.64	1.68	86.38	55.25	22.94	2.19	82.77	15.33	1.56	1.22
P	Hut-78	1.90	1.69	ns	ns	ns	ns	ns	ns	65.99	11.71	ns	ns	39.11	3.81	ns	ns

For each cell line (Origin; P: primary tumor, M: metastasis) mean percentage (%) and mean fluorescence index (MFI; calculated as the ratio between the mean fluorescence of the samples stained with the mAb anti-receptor and their correspondent isotypic control) of cells which significantly expressed (p < 0.05) chemokine ligands intracellularly are shown (ns: not significant expression).

### Secretion of chemokines CXCL9, CXCL10, CXCL11, CXCL12, CCL19, CCL21, CCL27 and CCL28 in human melanoma cell lines

CXCL10 was the only chemokine secreted in the studied melanoma cell lines. This chemokine was secreted in low concentrations by A375 and SK-Mel2 (40 pg/ml and 38 pg/ml, respectively). All melanoma cell lines secreted the control chemokines IL-8 and Gro (although at different amounts, Table 5).

**Table 5:** Secretion of chemokines

***Origin***	***Cell line***	**GRO**	**IL-8**	**CXCL10**
P	IPC-298	936	821	§
P	Mel-Juso	3939	6663	§
P	Mel-HO	439	274	§
P	IGR-39	117	787	§
P	WM-115	341	7614	§
M	A-375	4091	5234	40
M	MeWo	1234	235	§
M	SK-Mel28	197	212	§
M	Malme-3 M	217	43	§
M	Sk-Mel2	1956	7457	38
M	WM-266-4	6419	346	§
M	IGR-37	2740	117	§
M	Mel-RC08	1921	4296	§
P	Hut-78	§	§	550

For each cell line (Origin; P: primary tumor, M: metastasis) the mean concentration (pg/ml) of secreted chemokines is shown (§ concentration below the lower limit of detection).

### Immunocytochemistry

An immunocytochemical analysis of the cell lines was performed to check the intracellular presence of the chemokine receptors and chemokines (with the exception of CCL28). The results confirm the results obtained by flow cytometry, with staining of all receptors and chemokines, with the exception of CCL10. Some representative examples are shown in Figure 2 (C and D) and Figure 3 (C and D).

### Surface and intracellular expression of chemokines and their receptors in WM-115 and WM-266.4 cell lines after xenotransplantation

Tumors obtained from the xenotransplanted WM-266.4 cell line grew faster than those obtained from the WM-115 cell line. The growth of the former was noticed after 10 days of inoculation while the growth of the latter was noticed after 30 days of inoculation. After tumor disaggregation, a subset of cells was used directly for quantification of the expression of chemokines and their receptors by flow cytometry and another subset was cultured for a few passages for posterior quantification, in order to compare them with the original cell lines. When cultured, the cells exhibited some variability in the morphology and were slightly different from the original cell lines.

#### Surface expression of chemokine receptors

When compared with the original cell lines, the WM-115-X and WM-266-X tumors obtained after xenotransplantation, as well as the cell lines derived from them (WM-115-CX and WM-266-CX) had similar patterns of cell surface expression of receptors, that is, minute or no expression was observed, without significant differences with the original cell lines.

#### Intracellular expression of chemokine receptors

**Figure 4:**
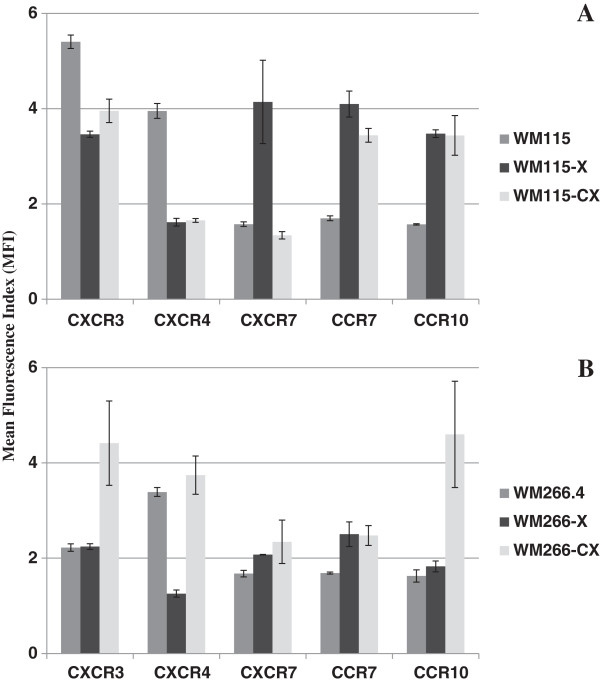
**Intracellular expression of chemokine receptors in WM-115 and WM-266.4 cell lines after xenotransplantation.** Comparison between mean fluorescence index (MIF, ± standard error) values of intracellular chemokine receptors in the original melanoma cell lines WM-115 **(A)** and WM-266.4 **(B)** and the tumors and cell lines obtained after xenotransplantation.

When compared with the original cell line, the WM-115-X xenografts, as well as the derived WM-115-CX cell lines, showed a substantial increase in the expression of CCR7 and CCR10, a considerable decrease in the expression of CXCR4 and a slight but significant decrease in the expression of CXCR3. CXCR7 showed an increased expression in the WM-115-X xenografts that was not observed in the derived WM-115-CX cell lines that were not significantly different from the original cell line (Table 6, Figure 4A). WM-266-X xenografts showed a significant decrease of CXCR4 and significant increases of CXCR7 and CCR7, when compared with the original cell line, while the WM-266-CX derived cell lines presented increases of CXCR3, CCR7 and CCR10 with respect to the original cell line (Table 6, Figure 4B).

**Table 6:** Intracellular expression of chemokine receptors after xenotransplantation

	***CXCR3***		***CXCR4***		***CXCR7***		***CCR7***		***CCR10***	
	***%***	***MFI***	***%***	***MFI***	***%***	***MFI***	***%***	***MFI***	***%***	***MFI***
WM-115	88.59	5.40	92.45	3.95	4.97	1.57	20.16	1.70	13.67	1.57
WM-115-X	59.84	3.46	14.10	1.62	58.89	4.14	75.32	4.10	66.90	3.47
WM-115-CX	76.11	3.95	12.14	1.65	5.44	1.34	82.03	3.44	81.90	3.44
WM-266	74.08	2.22	96.26	3.39	21.53	1.68	40.16	1.69	24.00	1.63
WM-266-X	28.19	2.24	6.71	1.26	13.96	2.08	56.31	2.50	28.54	1.83
WM-266-CX	91.65	4.41	94.17	3.74	53.67	2.34	72.95	2.48	88.62	4.60

Mean percentage (%) and Mean fluorescence index (MFI; calculated as the ratio between the mean fluorescence of samples stained with the mAb anti-receptor and correspondent isotypic control) of cells which significantly expressed (p < 0.05) chemokine receptors intracellularly. WM-115 and WM-266 represent the mean values of the original cell lines; WM-115-X and WM-266-X represent the mean values of the different xenografts; WM-115-CX and WM-266-CX represent the mean values of the different cell lines obtained after culturing the xenografts.

#### Intracellular expression of chemokines

**Figure 5:**
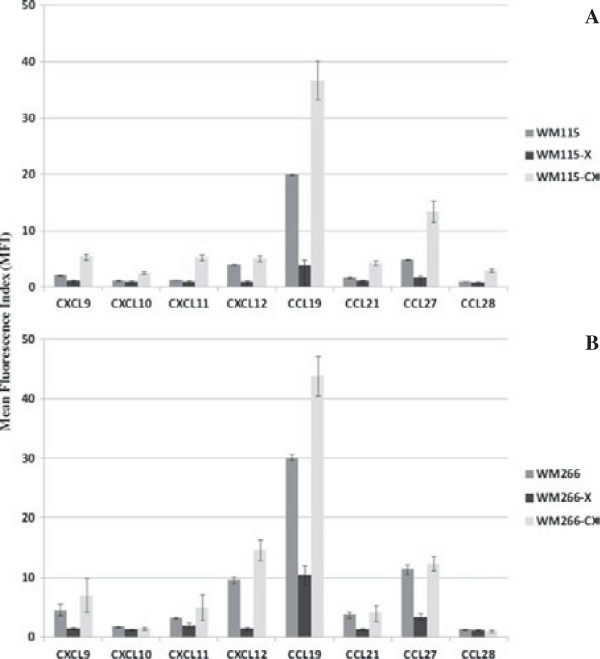
**Intracellular expression of chemokines in WM-115 and WM-266.4 cell lines after xenotransplantation.** Comparison between mean fluorescence index (MIF, ± standard error) values of intracellular chemokines in the original melanoma cell lines WM-115 **(A)** and WM-266.4 **(B)** and the tumors and cell lines obtained after xenotransplantation.

In WM-115-X xenografts a general decrease of chemokine expression is observed to levels similar to negative controls for most of the studied chemokines with respect to the initial WM-115 cell line, as can be detected by mean fluorescence index (Table 7). In WM-115-CX lines derived after xenotransplantation an increased expression of all chemokines is observed, when compared with the original cell line (Table 7, Figure 5A). In WM-266-X xenografts, a general decrease of chemokine expression is also observed when compared with WM-266.4 cell line, but that does not reach negative control values. The WM-266-CX cell lines derived from the xenografts only show significant increased expression of CXCL12 and CCL19 (Table 7, Figure 5B) with respect to the original cell line.

**Table 7:** Intracellular chemokine expression after xenotransplantation

	***CXCL9***		***CXCL10***		***CXCL11***		***CXCL12***		***CCL19***		***CCL21***		***CCL27***		***CCL28***	
	***%***	***MFI***	***%***	***MFI***	***%***	***MFI***	***%***	***MFI***	***%***	***MFI***	***%***	***MFI***	***%***	***MFI***	***%***	***MFI***
WM-115	28.31	2.06	0.46	1.12	2.66	1.24	81.77	3.96	98.79	20.00	15.55	1.65	94.06	4.89	ns	0.94
WM-115-X	ns	1.10	ns	0.90	ns	0.96	0.30	0.94	50.38	3.80	ns	1.05	22.39	1.62	ns	0.97
WM-115-CX	50.39	5.36	4.33	2.54	15.24	5.25	79.83	5.08	98.64	36.76	14.46	4.24	98.17	13.36	3.51	2.98
WM-266	71.84	4.57	15.19	1.74	46.26	3.24	98.24	9.56	98.58	30.17	60.21	3.71	98.50	11.38	1.14	1.25
WM-266-X	2.12	1.46	2.53	1.28	22.73	1.93	3.81	1.42	81.95	10.43	2.38	1.33	54.41	3.39	1.25	1.19
WM-266-CX	67.84	7.02	3.72	1.41	37.75	4.98	95.52	14.66	98.10	43.82	50.06	4.00	97.12	12.36	0.29	0.97

Mean percentage (%) and mean fluorescence index (MFI; calculated as the ratio between the mean fluorescence of samples stained with the mAb anti-receptor and correspondent isotypic control) of cells which significantly expressed (p < 0.05) chemokine ligands intracellularly. WM-115 and WM-266 represent the mean values of the original cell lines; WM-115-X and WM-266-X represent the mean values of the different xenografts; WM-115-CX and WM-266-CX represent the mean values of the different cell lines obtained after culturing the xenografts.

## Discussion

It has been previously demonstrated that chemokine receptors allow the directed migration towards specific organs [21]. Moreover, the receptors CXCR3, CXCR4, CCR7 and CCR10 have been implicated in the process of metastasis in melanoma, based on studies with animals [12,13]. Some of them, namely CXCR4, were also associated with metastasis in other types of neoplasms like breast, prostate, ovarian, colon and lung cancers [21]. CXCR7 is a chemokine receptor that shares ligands CXCL11 and CXCL12 with CXCR3 and CXCR4, respectively and that has recently been found to mediate CXCL12-induced migration in normal human epidermal melanocytes [22].

Most studies that analyze these chemokine receptors at the protein level in melanomas have been performed using mainly immunohistochemistry and western blotting techniques, which do not permit the correct evaluation of cell surface expression. Gene expression studies at the mRNA level have shown that human melanoma cell lines express CXCR4 and also receptors CCR10 and CCR7 which could be implicated in the frequent metastasis of melanoma to skin and lymph nodes, respectively [21]. However, these studies do not imply either the presence of functional receptors at the cell surface. On the other hand, few studies have determined the production by human melanoma cells of the chemokines that interact with these receptors. Using immunohistochemical techniques, a correlation has been found between T immunoreactive cells and the expression of CCR10 and its ligand CCL27 in cutaneous melanocytic lesions [14]. Expression of both receptor and chemokine was found in human melanoma cells. Their results suggest that in human melanomas CCR10 and CCL27 may act on the ability of neoplastic cells to grow, invade tissue, disseminate to lymph nodes and to escape the host immune response. Recently, immunohistochemical expression of CXCR4, CCR7 and CCR10 and their ligands has been described in tumor cells from primary and metastatic melanomas. The CXCL12-CXCR4 and CCL27-CCR10 ratios quantified by real time RT-PCR were found to be significantly higher in thin than in thick primary melanomas, and inversely associated with the development of distant metastasis [23]. Although these studies demonstrate the production of chemokines by the tumor cells they do not necessarily indicate their secretion from the cell. In this study we have determined the surface and intracellular expression of chemokine receptors, as well as the intracellular chemokine expression using flow cytometry and the chemokine secretion to the extracellular medium using commercial multiplex kits, to ascertain the extracellular chemokine-ligand interaction in human melanoma cell lines. Flow cytometry is one of the methods available for immunophenotyping (i.e. cellular phenotyping using antibodies) which allows working with live cells and, therefore, to analyze cell surface expression of proteins like receptors [24].

We have evaluated, using flow cytometry, the expression of the chemokine receptors CXCR4, CXCR3, CXCR7, CCR7 and CCR10, at cell surface and intracellular levels, for thirteen human melanoma cell lines. We detected surface expression of CXCR3 in the cell lines IPC-298, MEL-HO, IGR39, WM-115, SK-Mel 2, and IGR-37, surface expression of CXCR7 in the cell lines Mel-Juso, MEL-HO, IGR-39, SK-Mel 2, and IGR-37, and surface expression of CCR10 in the cell lines IPC-298, Mel-Juso, IGR-39, WM-115 and SK-Mel2. However, in all cases this expression was only detected in a small subpopulation of cells (less than 2%). Our results differ from a study that describes functional plasma membrane CXCR4 in the cell lines MeWo and A375 [15].

All cell lines significantly expressed all the receptors intracellularly, although there was significant variability in the pattern of expression between the different cell lines. The intracellular expression of chemokine receptors, in other tumors, has been shown to be correlated with metastasis directed to lymph nodes and with a bad prognosis (e.g., CXCR4 in breast cancer [25]; in lung cancer [26]; in colon cancer [27]). In the case of hepatocellular carcinoma the intracellular expression of CXCR4 with lack of its expression at the cell surface and lack of response to its ligand CXCL12 has been reported [28].

In normal cells, CXCR7 protein expression in human differentiated neurons is mostly limited to the intracellular compartment with little to no expression on the plasma membrane [29]. In the case of multipotent mesenchymal stem cells (MSC) intracellular expression, at protein level, of chemokine receptors CCR1, CCR3, CXCR3, CXCR4 and CXCR6 has been found [30]. However, the surface expression of these chemokine receptors was much more restricted with only one of the chemokine receptors (CXCR6) displaying a strong signal.

A major feature of solid tumor microenvironment is hypoxia, i.e. decreased availability of oxygen [31]. Indeed, there are studies which show an increase of chemokine receptor expression in hypoxic conditions. For example, an increase in CXCR4 surface expression in the two human breast cancer cell lines, MDA-MB-231 and MCF7, following exposure to hypoxia resulted in a significant increase in migration and invasion in response to SDF1-alpha in vitro [32]. However, after submitting the primary cell lines WM-115 and IGR-39, and the metastatic cell lines WM-266.4 and IGR-37 to hypoxic conditions, we still failed to find an increase in cell surface expression of the chemokine receptors studied (results not shown).

Chemokine receptors form part of the family of G-protein coupled receptors. The appropriate delivery of chemokine receptors to the cell surface to allow receptor -ligand interactions, and their subsequent retrieval from the plasma membrane are of fundamental importance for the regulation of their activity [3,33]. Both during and subsequent to synthesis, chemokine receptors undergo a process of maturation before reaching the cell membrane. They must be properly inserted into the cell membrane, achieve their correct folding while still resident at the endoplasmic reticulum, traverse from the cis- to the trans-Golgi while undergoing modification, and finally be targeted to the plasma membrane where they attain residence as mature proteins. In order for a chemokine receptor to transduce an extracellular signal it must both traffic to and be retained at the cellular surface to allow for receptor- ligand interaction. Multiple proteins not involved in the signal transduction cascade have been identified which stabilize receptor surface expression [34]. Post-translational modifications can also alter surface expression of the receptor. In neuroblastoma, CXCR4 surface expression requires ubiquination and oligomerization of the receptor [35]. Finally, factors involved in the endocytic and recycling pathways could also affect the amount of receptor expressed at the plasma membrane [3]. For instance, CCR7 recycling to the cell surface has been found to be dependent on ubiquitination of the receptor [36].

In this work we have also quantified by flow cytometry the intracellular protein expression of the chemokines which activate each of the receptors studied: CXCL9, CXCL10 (CXCR3), CXCL11 (CXCR3 and CXCR7), CXCL12 (CXCR4 and CXCR7), CCL19, CCL21 (CCR7) and CCL27, CCL28 (CCR10). We found a certain pattern in their expression. Most chemokines were expressed in all cell lines. However, chemokines CXCL10 and CCL28 had a low or null expression in most of the cell lines (Table 4).

We also analyzed the secretion of chemokines in the culture medium of all cell lines. From all the chemokines studied, the only chemokine secreted by the melanoma cell lines was CXCL10. This chemokine was secreted in low concentrations by A375 and SK-Mel-2. As a positive control we also quantified the secretion of the IL-8 and Gro chemokines that are produced by human melanoma [37].

There is evidence, based on experiments designed to avoid HIV-1 infection [38,39], that expression of genetically modified chemokines (intrakines) with an added endoplasmic reticulum retention signal are able to avoid surface expression of their chemokine receptors, by interacting with the nascent chemokine receptors and retaining them in the endoplasmic reticulum. This procedure has been extended to other chemokine receptors not involved in HIV infection [40,41]. The transfection with the native chemokine without the endoplasmic retention signal also inhibited viral entry as demonstrated by the inhibitory effects on the syncytium formation [38], suggesting that the native chemokine can also prevent the transport of the receptor to the cell surface. This early interaction of chemokines with their chemokine receptors could alter post-translational processes, like glycosylations [42], or interactions with escort proteins that have been found necessary for trafficking to the plasma membrane and for expression of the proteins on the cell surface in other members of the family of G-protein-coupled receptors to which chemokine receptors belong [43,44], therefore resulting in intracellular accumulation of chemokine receptors and interacting chemokines. Interestingly, the only chemokine that was found to be secreted in two cell lines in our study, CXCL10, shows a minimal or no intracellular expression in the melanoma cell lines a fact that could reflect that it does not interact intracellularly with its receptor and therefore is not accumulated within the cell.

Tumor microenvironment originates in the interactions between malignant and non-transformed cells. Intercellular communication is driven by a complex network of cytokines, chemokines, growth factors, and inflammatory and matrix enzymes [45]. Chemokines and their receptors are important in cancer for cell trafficking into and out of the tumor microenvironment, and chemokines made by malignant and stromal cells contribute to the tumor-associated leukocyte component, to angiogenesis and to the generation of fibroblast stroma. Changes in the tumor microenvironment such as hypoxia, as we mentioned previously, or different molecular factors can alter the expression of chemokines and their receptors in the malignant cells [46]. In order to provide an *in vivo* environment and stimuli to these established melanoma cell lines we xenografted the primary cell line WM-115 and the metastatic cell line WM-266.4 that were initially derived from the same patient [47], into nude mice. We obtained five different tumors from the primary cell line and six different tumors from the metastatic cell line (named WM-115-X and WM-266-X, respectively). Cells obtained from collagenase treatment of these tumors were analyzed directly by flow cytometry. There were no significant changes in expression of receptors at the cell surface, although it must be considered that the disaggregation procedure could influence the detection of the receptors at this level, as in the case of the cell lines these were detached solely using EDTA to avoid the effect of trypsin on the surface cell receptors. Intracellular receptor and chemokine content varies in the xenograft with respect to the original cell line. In WM-115-X there is a significant reduction of CXCR3 and CXCR4, and a significant increase of CXCR7, CCR7 and CCR10, while in WM-266-X there is a significant decrease of CXCR4 and modest but significant increases in CCR7 and CCR10. The cell lines derived from the xenografts showed dynamic variations in the expression of intracellular chemokines and chemokine receptors when compared with the original cell lines. The changes in protein expression were different in the primary cell line with respect to the metastatic cell line. WM-115-CX showed a decreased expression of CXCR4 and CXCR3 together with an increased expression of CCR7 and CCR10, while WM-266-CX had an increased expression of CXCR3, CCR7 and CCR10 (Figure 4). However, cell surface expression of these receptors remained very low or inexistent in both cases. WM-115-CX showed a higher intracellular expression of all the tested chemokines, while WM-266-CX showed intracellular chemokine values that were similar to the original cell line, with the exception of CCL12 and CCL19 that show an increase (Figure 5).

A comparative analysis of global gene expression has been performed between human melanoma cell lines with different metastatic capacity and the xenografts obtained by their subcutaneous injection into immunocompromised mice [48], demonstrating extensive differential expression between both models. These variations can be due to selection of determined subpopulations with different tumorigenic capacity or to the effect of the different microenvironment within the nude mouse. Melanoma is a highly heterogeneous tumor that shows a high degree of plasticity. Recent evidence indicates that WM-266.4 xenografts contain a cell subpopulation expressing ABCB5 endowed with intrinsic chemoresistance [49]. ABCB5 has been described as a marker for tumor-initiating cells in human melanoma [50]. Although the nature and frequency of cancer stem cells in melanoma is controversial, it is likely that this model can be applied to human melanoma, and different surface markers have been proposed to define melanoma cell with tumor initiating capacity [51]. Studies performed on melanoma cells growing as melanospheres isolated selectively from attached cell lines, heterogeneously expressing different stem cell markers, more efficiently form tumors in immunocompromised mice than cell line [52], suggesting that various subpopulations can participate in the formation of the tumor. In our study a selection process seems to take place within the tumoral population in the xenograft, especially in the case of WM-115 derived from a primary tumor where the changes observed in the intracellular chemokine receptors that have been involved in tumoral growth and progression are maintained in the derived cell lines in culture medium. However, intracellular chemokine levels show a general decrease in the xenografts, being more pronounced in WM-115-X than in WM-266-X and that seems to be due to environmental causes, as these changes are not maintained in the derived cell lines.

## Conclusions

In conclusion, we find coexpression of chemokine receptors and their ligands in human melanoma cell lines. However, this expression is intracellular and receptors are not found at the cell membrane nor chemokines are secreted to the cell medium. The levels of intracellularly expressed chemokine receptors and their ligands show dynamic variations after xenotransplantation that differ depending on the origin of the cell line. These changes could affect cell trafficking assays that correlate in vitro and in vivo data. These results could also have implications in the studies that analyze chemokines or chemokine receptors expression in melanomas that do not ascertain the cell membrane location of chemokine receptors or the secretion of chemokines to the extracellular medium.

## Competing interests

The authors declare that they have no competing interests.

## Authors’ contributions

SP, AMR, RGB, CM and RCC designed the study. SP carried out the flow cytometry and chemokine secretion experiments, did the statistical analysis and drafted the manuscript. RGB, TSN, LT carried out the immunocytochemistry assays and performed the xenografts. All authors interpreted the results, read and approved the final manuscript.

## Pre-publication history

The pre-publication history for this paper can be accessed here:

http://www.biomedcentral.com/1471-2407/14/118/prepub
